# Human Papillomavirus Infection and Anxiety: Analyses in Women with Low-Grade Cervical Cytological Abnormalities Unaware of Their Infection Status

**DOI:** 10.1371/journal.pone.0021046

**Published:** 2011-06-16

**Authors:** Candice Y. Johnson, Linda Sharp, Seonaidh C. Cotton, Cheryl A. Harris, Nicola M. Gray, Julian Little, in collaboration with the TOMBOLA Group

**Affiliations:** 1 Department of Epidemiology, Rollins School of Public Health, Emory University, Atlanta, Georgia, United States of America; 2 National Cancer Registry Ireland, Cork, Ireland; 3 Health Services Research Unit, University of Aberdeen, Aberdeen, United Kingdom; 4 Department of Psychology and Psychosocial Oncology Program, The Ottawa Hospital Cancer Centre, Ottawa, Ontario, Canada; 5 Centre of Academic Primary Care, University of Aberdeen, Aberdeen, United Kingdom; 6 Canada Research Chair in Human Genome Epidemiology, Department of Epidemiology and Community Medicine, University of Ottawa, Ottawa, Ontario, Canada; St. Petersburg Pasteur Institute, Russian Federation

## Abstract

**Background:**

Women testing positive for human papillomavirus (HPV) infection experience increased levels of anxiety that have been attributed to fears of stigmatization and developing cervical cancer. The objective of this study was to investigate the association between HPV infection and anxiety in women who were unaware they had been tested specifically for HPV, to determine if any anxiety experienced by HPV-positive women could be due to causes other than learning of test results.

**Methods:**

This study was nested within a randomised controlled trial of management of women with abnormal cervical cytology conducted in the United Kingdom with recruitment between 1999 and 2002. At baseline, prior to having a sample taken for HPV testing, the results of which were not disclosed, women were assessed for anxiety using the Hospital Anxiety and Depression Scale and asked about fears of developing cervical cancer (“cancer worries”); this assessment was repeated at 12, 18, 24, and 30 months of follow-up. Logistic regression and generalized estimating equations were used for the cross-sectional (baseline) and longitudinal analyses, respectively.

**Results:**

Among the 2842 participants, there was no association between HPV status and anxiety among white women. Among non-white women, however, anxiety was less common among HPV-positive than HPV-negative women (adjusted odds ratio 0.41, 95% confidence interval 0.22 to 0.77). Among non-smokers, cancer worry was more common in HPV-positive than HPV-negative women; the opposite association was observed among ex-smokers.

**Conclusions:**

Associations between HPV status and anxiety may be explained by factors other than learning of test results and may vary by ethnicity and lifestyle factors.

## Introduction

Worldwide, an estimated 10% of women are infected with human papillomavirus (HPV) and with a lifetime risk of infection near 80%, HPV is considered to be one of the most common sexually transmitted infections [1], [2]. Of over 100 HPV types known to infect humans, about 15, most commonly types 16 and 18, have been causally linked with cervical cancer, and are classified as “high-risk” [3], [4]. Testing for high-risk HPV is being considered as an alternative or supplement to current cervical screening procedures, as it is a more sensitive test than cervical cytology for the detection of cervical intraepithelial neoplasia and few cases of cervical cancer are thought to occur in the absence of HPV infection [5], [6], [7]. However, there has been concern about widespread adoption of HPV testing since receipt of positive test results has been associated with psychological distress [8], [9].

Qualitative and quantitative studies have found that women testing positive for HPV experience anxiety, report feelings of stigmatization, and worry about disclosing test results to their partner, family, and friends [8], [9], [10]. Studies in women with low-grade abnormal cytology results, who may already have elevated anxiety from learning of these results, have reported similar findings. In these studies, anxiety, distress, and intrusive thoughts were also more common in HPV-positive women than those testing negative [8], [11]. In studies comparing women with normal and abnormal cytology, however, a greater difference in anxiety and psychological distress was observed between HPV-positive and HPV-negative women in the group with normal cytology than in those with abnormal results [9], [12].

Most studies reporting associations between HPV and anxiety have been conducted after women had received their test results and have focused on the positive test result as the only potential cause of anxiety [8], [9], [11]. In these studies, it has not been possible to separate the effects of learning of a positive test result from the effects of any baseline differences in anxiety between HPV-positive and HPV-negative women. Lifestyle and sociodemographic risk factors for HPV include age, ethnicity, socioeconomic status, and smoking status; these are characteristics also associated with anxiety [13], [14], [15], [16]. As such, it is possible that elevated anxiety following HPV testing is at least partially attributable to HPV-positive women having more risk factors for anxiety than those who are HPV-negative. Results consistent with this hypothesis have been suggested by the ARTISTIC trial, in which anxiety and psychological distress were observed to be more common in HPV-positive women than HPV-negative women even though women were unaware of their HPV test results, which had not been disclosed to them [12].

The aim of this study was to determine if adverse psychological effects experienced by HPV-positive women could be due to causes other than learning of test results. We examined whether anxiety and cancer worries were more common in HPV-positive than in HPV-negative women using data from a large, population-based sample of women undergoing management for low-grade abnormal cervical cytology, who had undergone HPV testing but were not aware of their test result. We also assessed whether the association between HPV status and anxiety differed by lifestyle or sociodemographic risk factors for HPV and anxiety.

## Methods

### Ethics statement

Ethics approval was obtained from the joint research ethics committee of NHS Grampian and the University of Aberdeen, the Tayside committee on medical research ethics, and the Nottingham research ethics committee. All participating women provided written informed consent.

### Participants

The study population consisted of women who participated in TOMBOLA (Trial Of Management of Borderline and Other Low-grade Abnormal smears), a randomised controlled trial to determine the most effective and efficient management for women with low-grade abnormal cervical cytology (borderline nuclear abnormalities or mild dyskaryosis, broadly equivalent to ASCUS [atypical squamous cells of undetermined significance] and LSIL [low grade squamous intraepithelial lesion] in the Bethesda system) [17], [18], [19], [20], [21], [22], [23]. Between 1999 and 2002, women in the Grampian and Tayside (Scotland) and Nottingham (England) areas aged 20–59 years undergoing routine cervical screening in primary care and who had had a recent routine cytology result classified as low-grade abnormal were invited to participate in the trial. Women who were pregnant or who had previously been treated for cervical lesions were excluded. After entry into the trial, women were randomised to receive either initial colposcopy or cytological surveillance (cytology repeated at 6-monthly intervals in primary care), and were followed for a three-year period.

### Outcome assessment: anxiety and cancer worry

Anxiety was the primary outcome of interest assessed by means of the anxiety subscale of the Hospital Anxiety and Depression Scale (HADS) [24]. Although the HADS was originally intended for use in hospital outpatients, it has since been shown to be valid for screening for anxiety and depression in primary care and community settings [25]. The instrument was administered during the TOMBOLA recruitment appointment at a hospital clinic, a median of approximately 10 weeks following the initial low-grade abnormal cytology result, and again at 12, 18, 24, and 30 months of follow-up by mailed questionnaire. Women were asked to respond to each question in relation to how they had been feeling over the previous week.

Cervical cancer worry was a secondary outcome of interest. Such worries are commonly reported in women undergoing follow-up for abnormal cervical cytology results [26]. Since 60% of women without anxiety in our series reported cervical cancer worry (Table 1), we therefore considered that cancer worry might be a more specific measure of psychological distress for this population of women. Cervical cancer worry was measured in the recruitment questionnaire by the question “Since getting my smear result I have been worried that I may have cervical cancer”, with choices of responses in the form of a Likert scale (strongly agree, moderately agree, slightly agree, slightly disagree, moderately disagree, strongly disagree) [26]. Mailed questionnaires were used to measure cancer worry after 12, 18, 24, and 30 months of follow-up.

**Table 1:** Characteristics of the 2842 participants with respect to HPV status, cancer worry, and anxiety at baseline.

		HPV status			Cervical cancer worry			Anxiety		
		N (column %)			N (column %)			N (column %)		
Variable	Level	Positive	Negative	p*	Yes	No	p*	Yes	No	p*
N (row %)		1164 (41.0)	1678 (59.0)		1938 (68.2)	904 (31.8)		1645 (57.9)	1197 (42.1)	
Cancer worry	Yes	352 (30.2)	552 (32.9)	0.13				978 (81.7)	960 (58.4)	<0.01
	No	812 (69.8)	1126 (67.1)					219 (18.3)	685 (41.6)	
HADS anxiety score	0–7	667 (57.3)	978 (58.3)	0.60	960 (49.5)	685 (75.8)	<0.01			
	≥8	497 (42.7)	700 (41.7)		978 (50.5)	219 (24.2)				
Trial arm	Colposcopy	575 (49.4)	855 (51.0)	0.41	991 (51.1)	439 (48.6)	0.20	613 (51.2)	817 (49.7)	0.42
	Cytological surveillance	589 (50.6)	823 (49.0)		947 (49.9)	465 (51.4)		584 (48.8)	828 (50.3)	
Trial centre	A	363 (31.2)	508 (30.3)	0.03	531 (27.4)	340 (37.6)	<0.01	331 (27.7)	540 (32.8)	0.01
	B	227 (19.5)	396 (23.6)		416 (21.5)	207 (22.9)		272 (22.7)	351 (21.3)	
	C	574 (49.3)	774 (46.1)		991 (51.1)	357 (39.5)		594 (49.6)	754 (45.8)	
Age	20–29	705 (60.6)	513 (30.6)	<0.01	793 (40.9)	425 (47.0)	<0.01	515 (43.0)	703 (42.7)	0.03
	30–39	276 (23.7)	478 (28.5)		557 (28.7)	197 (21.8)		342 (28.6)	412 (24.1)	
	40–49	138 (11.9)	468 (27.9)		421 (21.7)	185 (20.5)		247 (20.6)	359 (21.8)	
	50–59	45 (3.9)	219 (13.1)		167 (8.6)	97 (10.7)		93 (7.8)	171 (10.4)	
Ethnicity	White	1114 (95.7)	1606 (95.7)	1.00	1846 (95.3)	874 (96.7)	0.08	1141 (95.3)	1579 (96.0)	0.39
	Non-white	50 (4.3)	72 (4.3)		92 (4.8)	30 (3.3)		56 (4.7)	66 (4.0)	
Smoking status	Non-smoker	527 (45.3)	827 (49.3)	<0.01	897 (46.3)	457 (50.6)	0.05	510 (42.6)	844 (51.3)	<0.01
	Ex-smoker	154 (13.2)	339 (20.2)		706 (36.4)	289 (32.0)		485 (40.5)	510 (31.0)	
	Current smoker	483 (41.5)	512 (30.5)		335 (17.3)	158 (17.5)		202 (16.9)	291 (17.7)	
Carstairs deprivation index	Highest quintile (least deprived)	153 (13.1)	258 (15.4)	<0.01	283 (14.6)	128 (14.2)	0.09	157 (13.1)	245 (15.4)	<0.01
	2^nd^ quintile	192 (16.5)	346 (20.6)		353 (18.2)	185 (20.5)		200 (16.7)	338 (20.6)	
	3^rd^ quintile	167 (14.4)	294 (17.5)		304 (15.7)	157 (17.4)		196 (16.4)	265 (16.1)	
	4^th^ quintile	314 (27.0)	414 (24.7)		490 (25.3)	238 (26.3)		319 (26.7)	409 (24.9)	
	Lowest quintile (most deprived)	338 (29.0)	366 (21.8)		508 (26.2)	196 (21.7)		325 (27.2)	379 (23.0)	

*p-value from chi-square test.

### Exposure assessment: HPV testing

Details of the HPV testing have been described elsewhere [17]. Briefly, cervical samples were obtained from participants at the TOMBOLA recruitment appointment following administration of the sociodemographic (see below) and psychosocial questionnaires. Women were told that the cervical swab would be used for additional testing, the results of which they would not receive. They were not told that the swabs would be used for testing HPV infection. DNA was extracted from the swabs using a QIAamp® DNA Mini Kit (Qiagen UK) and amplified using real-time polymerase chain reaction. Women were classified as HPV-positive at baseline if they were infected with any of fourteen high-risk HPV types (HPV16, 18, 31, 33, 35, 39, 45, 51, 52, 56, 58, 59, 66, or 68). The results of the HPV test were not disclosed to the participants or to health professionals involved in their care.

### Covariate assessment

Information on sociodemographic characteristics was collected at study entry during the TOMBOLA recruitment appointment and consisted of questions concerning the participants' ethnicity, reproductive history, education, smoking, and physical activity.

### Statistical analysis

The study population was restricted to the 3258 women who completed at least half of the HADS anxiety subscale at baseline (recruitment) (i.e., they had completed 4 or more of the 7 questions). Best subset regression was used to impute scores for women who had answered at least half, but not all, of the anxiety subscale questions at any time point (n = 27, 24, 25, 18, 10 at baseline and 12, 18, 24, and 30 months of follow-up, respectively). Women (n = 416) with baseline values missing for cancer worry (n = 36), HPV status (n = 351), or any covariates included in the multivariable analysis (n = 29) were excluded, leaving 2842 in the final analysis. Outcomes were dichotomized, with significant anxiety defined as a score of 8 or greater on the HADS anxiety subscale (HADS anxiety subscale range: 0–21) [24], [27] and cervical cancer worry defined as any positive response to the question on cervical cancer worry (strongly, moderately, or slightly agree). HPV status was categorised as high-risk positive or negative.

Cross-sectional analyses were performed using logistic regression to estimate odds ratios (OR) and 95% confidence intervals (CI) for associations at baseline between HPV status (exposure) and (a) anxiety or (b) cancer worry. Generalized estimating equations with an autoregressive correlation structure were used to take into account the longitudinal nature of the data when outcome measurements from baseline and 12, 18, 24, and 30 months of follow-up were included. To explore the impact of using a defined cut-off for the definition of significant anxiety, a sensitivity analysis was undertaken. The analyses were repeated treating HADS anxiety score as a continuous variable using linear regression and generalized estimating equations for the cross-sectional and longitudinal analyses, respectively. The analyses were also repeated without imputation for missing HADS anxiety scores. In each analysis, all variables aside from the outcomes (anxiety and cancer worry) were assumed to be time-invariant.

Potential confounders or effect measure modifiers considered in the models included: trial centre (A, B, C), trial arm (colposcopy, cytological surveillance), age at recruitment smear (20–29, 30–39, 40–49, 50–59), ethnicity (white, non-white), smoking (never, ex-, or current smoker), and neighbourhood deprivation (quintiles of the Carstairs Index [28]). To assess effect measure modification, cross-product terms were fitted between exposure (HPV status) and each covariate, with terms retained in the final model if the likelihood ratio test (cross-sectional analysis) or score test (longitudinal analysis) was statistically significant (i.e., p<0.05). All analyses were performed in SAS version 9.2 (SAS Institute, Cary, NC).

## Results

### Participant characteristics

At study entry, 1164 (41%) women were HPV-positive (Table 1). Over half of participants (58%) were classified at baseline as having significant anxiety and slightly more than two-thirds (68%) reported cervical cancer worry. In univariate analysis, trial centre, age, and smoking status were associated with each of HPV infection, anxiety, and cancer worry.

### HPV status and anxiety

In the cross-sectional analysis, no association was found between HPV status alone and anxiety (crude OR 1.04, 95% CI 0.90–1.21). In the fully-adjusted model, an interaction term between HPV status and ethnicity remained (p = 0.03). Anxiety was equally likely in HPV-positive and HPV-negative white women (adjusted OR 1.02, 95% CI 0.86–1.20), but was less likely in HPV-positive than HPV-negative non-white women (adjusted OR 0.42, 95% CI 0.20–0.90). Similar results were found in the longitudinal analysis (Table 2). When analyses were repeated without imputation of missing data, the results did not change (data not shown).

**Table 2:** Associations between HPV status and anxiety by ethnicity, and HPV status and cancer worry by smoking status.

Outcome	Estimate	Cross-sectional OR (95% CI)*	p for interaction†	Longitudinal OR (95% CI) ‡	p for interaction†
Anxiety	Overall estimate	0.98 (0.83–1.15)	0.03	0.99 (0.86–1.14)	<0.01
	White women	1.02 (0.86–1.20)		1.03 (0.90–1.18)	
	Non-white women	0.42 (0.20–0.90)		0.41 (0.22–0.77)	
Cancer worry	Overall estimate	1.16 (0.98–1.38)	<0.01	1.17 (1.05–1.31)	0.02
	Non-smokers	1.55 (1.21–1.98)		1.36 (1.16–1.59)	
	Current smokers	0.84 (0.63–1.11)		0.97 (0.80–1.16)	
	Ex-smokers	0.46 (0.25–0.84)		0.69 (0.46–1.02)	

*Odds ratio (OR) and 95% confidence interval (CI) for anxiety in HPV-positive (compared to HPV-negative) women at baseline, adjusted for trial centre, trial arm, age, smoking status, and Carstairs Index.

†p-value is for cross-product term in the regression model.

‡Odds ratio and 95% confidence interval for anxiety at baseline and over course of follow-up (12, 18, 24, 30 months) in HPV-positive (compared to HPV-negative) women where HPV status was determined at baseline, adjusted for trial centre, trial arm, age, ethnicity, and Carstairs Index.

Results from the sensitivity analyses with HADS anxiety score considered as a continuous variable were similar. No association was observed between HPV status and HADS anxiety score (cross-sectional: β = 0.23, 95% CI −0.11 to 0.56, longitudinal: β = 0.19, 95% CI −0.10 to 0.49). Unlike the analysis with dichotomized HADS score, no interaction term remained in the final adjusted model. Although the interaction term between HPV status and ethnicity did not reach formal statistical significance (p = 0.13), there was a weak positive association between HPV status and HADS anxiety score in white women while in non-white women, HPV infection appeared to be associated with lower anxiety scores (cross-sectional: β = −0.52, 95% CI −2.13 to 1.08, longitudinal: β = −1.05, 95% CI −2.42 to 0.31).

### HPV status and cancer worry

Both the cross-sectional and longitudinal analyses suggested weak associations between HPV status and cancer worry, with cancer worry more likely among HPV-positive women than HPV-negative women (cross-sectional: crude OR 1.13, 95% CI 0.96–1.33, longitudinal: crude OR 1.25, 95% CI 1.12–1.39). In the fully adjusted analysis, all interaction terms were removed from the model except that between HPV and smoking status (cross-sectional: p<0.01, longitudinal: p = 0.02). Both the cross-sectional and longitudinal analyses suggested that among non-smokers cancer worry was more common in HPV-positive women than HPV-negative women (Table 2). The association was not apparent in current smokers, while in ex-smokers the opposite association was seen, with cancer worry more common in HPV-negative than HPV-positive women.

## Discussion

Differences in the association between anxiety and HPV status were observed by ethnicity, with anxiety less common in HPV-positive compared to HPV-negative non-white women, while cancer worry was more common among HPV-positive women, but only among non-smokers. In the total population no association between HPV status and anxiety was observed, in contrast to previously reported findings from the ARTISTIC trial [12].

A major strength of this study was that participants were assessed for anxiety prior to their HPV test and were not told specifically that they were receiving such a test, and were not told their results. As a result, receipt of positive test results can be excluded as an explanation for anxiety or cancer worry experienced by HPV-positive women. It is possible that a few women knew their HPV status from previous tests, but it seems unlikely that this would substantially affect results given the low awareness of HPV and its relation to cervical cancer at the time this study was conducted [29]. The results suggest that something other than knowledge of the HPV results is influencing psychological wellbeing.

Reasons for the observed differences in the associations by ethnicity and smoking status are not immediately obvious, but there are several possible explanations. Pre-existing or long-term anxiety share risk factors with HPV infection, and it is possible that these associations may differ between strata of these risk factors or exist only in certain segments of the population. Information on risk factors related to sexual behaviours which might also correlate with anxiety, such as number of sexual partners and age at first intercourse, were not available in this study to further investigate this hypothesis [13]. Second, the prevalence, presentation, and response to treatment of anxiety are recognized to differ substantially by ethnicity, and these differences are hypothesized to have both cultural and biologic explanations [14], [15], [30]. These cultural and biologic determinants of anxiety may explain the observed differences between HPV and anxiety. A third possibility is that there are biologic mechanisms responsible for the association between HPV infection and anxiety, and these mechanisms differ by ethnicity or behaviour. An increasing body of evidence suggests that viral infections may be able to affect mood and behaviour [31], [32], [33]. It has been noted that some cancer patients treated with interferon therapy experience symptoms of anxiety and depression, suggesting that when the immune system produces these same signalling molecules during immune response to infection, similar behavioural symptoms may be possible [33]. It is unclear whether this sort of mechanism could provide an explanation for the association between HPV and psychological distress varying by ethnicity or smoking, but further investigation is warranted on the basis of these findings. A fourth, and perhaps likely, explanation for these results is that of chance. There were no specific *a priori* hypotheses regarding effect measure modification by smoking or ethnicity; in particular, with a small number of non-white women in the study (n = 122), investigation of this association in populations with larger proportions of non-white women would be beneficial.

Given the transient nature of most HPV infections [34], availability of HPV status at a single point in time is a limitation of this study. It would be unlikely that most women who were HPV-positive at baseline would remain so for the length of the three-year follow-up. HPV testing was also conducted at the end of the follow-up period, approximately 3 years post-recruitment. In the subgroup of 1639 women participating in TOMBOLA who had HPV status determined both at enrolment and at exit, 215 (13%) were HPV-positive at both time points. We have not conducted further analysis on this point, because not all of this subgroup had been eligible to participate in the psychosocial assessment. It is conceivable that if biologic effects of infection on anxiety exist, they would be most likely to be observed in women with persistent, rather than transient, HPV infection. A further limitation of the study is potential inability to detect weak effects on anxiety, since anxiety may have been initially elevated among participants due to the previous abnormal cytology result that was a condition of study eligibility [35]. Results from the ARTISTIC trial have suggested that while psychosocial distress and anxiety are elevated in HPV-positive compared to HPV-negative women with negative cytology, there is less of a difference observed in women with low-grade abnormal cytology [12]. The raised anxiety in women with low-grade abnormal cytology results decreases following further investigation and intervention [36]. This would suggest that the longitudinal analysis may be better suited to detecting longer-term anxiety not attributable to initial cytology results; in fact, the results from cross-sectional and longitudinal analyses were similar.

In the total study population, anxiety and cervical cancer worry were no more common in HPV-positive than HPV-negative women with low-grade abnormal cervical cytology who were unaware of their HPV status. Secondary analyses from this study suggest differences in associations between HPV status and anxiety and cervical cancer worry by ethnicity and smoking status. Explanations for these observed associations remain unclear and may be due to chance; however, it is possible that factors other than knowledge of test results, such as ethnicity and smoking status, may be important in explaining occurrence of anxiety and other adverse psychosocial outcomes in this population.
